# 12-Week Exercise Training, Independent of the Type of Exercise, Attenuates Endothelial Ischaemia-Reperfusion Injury in Heart Failure Patients

**DOI:** 10.3389/fphys.2019.00264

**Published:** 2019-03-15

**Authors:** Dick H. J. Thijssen, Nathalie M. M. Benda, Thijs P. Kerstens, Joost P. H. Seeger, Arie P. J. van Dijk, Maria T. E. Hopman

**Affiliations:** ^1^Department of Physiology, Radboud University Medical Center, Radboud Institute for Health Sciences, Nijmegen, Netherlands; ^2^Research Institute for Sport and Exercise Sciences, Liverpool John Moores University, Liverpool, United Kingdom; ^3^Department of Cardiology, Radboud University Medical Center, Radboud Institute for Health Sciences, Nijmegen, Netherlands

**Keywords:** exercise training, preconditioning, physical fitness, cardiovascular function, flow-mediated dilation

## Abstract

**Introduction:** Reperfusion is required to salvage ischaemic tissue, but also causes further damage (i.e., ischaemia/reperfusion-injury). Heart failure patients reveal exaggerated ischaemia/reperfusion-injury, whilst traditional ischaemic preconditioning cannot prevent ischaemia/reperfusion-injury. Exercise training may be a more powerful preconditioning stimulus, especially high-intensity interval training given the similarities with ischaemic preconditioning. Therefore, we examined the impact of 12-week continuous training vs. high-intensity interval training on brachial artery endothelial ischaemia/reperfusion-injury in heart failure patients New York Heart Association-class II-III.

**Methods:** Twenty heart failure patients (male:female 19:1, 64 ± 8 years, ejection fraction 38 ± 6%) were allocated to 12-weeks of high-intensity interval training (10^∗^1-min 90% maximal workload – 2.5-min 30% maximal workload) or continuous training (30-min 60–75% maximal workload). Before and after the intervention, we measured brachial artery endothelial function with flow-mediated dilation (FMD) before and after ischaemia/reperfusion (5-min ischemic exercise, 15-min reperfusion).

**Results:** Ischaemia/reperfusion caused a significant decline in FMD (continuous training (*n* = 10): 5.2 ± 2.5 to 3.4 ± 1.6%, high-intensity interval training (*n* = 10): 5.3 ± 2.6 to 3.5 ± 1.6%, *P* = 0.01), which was not different between groups (*P >* 0.05). Training improved maximal workload and fitness (*P* < 0.05), with no differences between groups (*P >* 0.05). Exercise training did not alter FMD (*P >* 0.05), whilst ischaemia/reperfusion did not impair FMD after exercise training (continuous training: 4.8 ± 3.0 to 4.2 ± 2.3%, high-intensity interval training: 4.7 ± 2.5 to 3.8 ± 2.3%, *P >* 0.05). No changes were found in FMD before or after ischaemia/reperfusion after 12-weeks in controls (*n* = 9).

**Conclusion:** We found that 12-week exercise training in heart failure patients mitigated endothelial ischaemia-reperfusion injury, an effect independent of the type of exercise. These changes may contribute to the cardioprotective effects of exercise training, whilst our findings highlight the potency of exercise as a preconditioning stimulus.

## Introduction

The prevalence of heart failure (HF) is increasing, and is characterized by a low 5-year survival of 35–55% (Bleumink et al., 2004). One potential reason for this poor prognosis may relate to ischaemia-reperfusion (IR)-injury. Although reperfusion is a common and effective strategy to restore blood flow to ischaemic (cardiac) tissue (Piccolo et al., 2015), this paradoxically causes significant additional damage (i.e., IR-injury) to the endothelium (Yellon and Hausenloy, 2007). Attenuating the deleterious effects of IR is therefore of utmost importance to further improve outcomes after myocardial infarction. Previous work in rats (Murray et al., 2006) and recently work from our group in humans (Seeger et al., 2016) revealed that HF is associated with exaggerated endothelial IR-injury. This highlights the need to explore strategies to attenuate endothelial IR-injury in HF patients.

Ischemic preconditioning (IPC) (i.e., short repetitive episodes of non-injurious ischemia and reperfusion) represents a potent strategy to reduce the severity of endothelium IR-injury (Murry et al., 1986). However, clinical trials using IPC have revealed somewhat disappointing results (Heusch, 2013), which may relate to the interaction between cardiovascular disease and the efficacy of IPC (van den Munckhof et al., 2013; Ferdinandy et al., 2014). Indeed, preclinical studies (Miki et al., 2000; Ghosh et al., 2001; Andersen et al., 2013) and *in vivo* work in humans (Seeger et al., 2016) revealed that HF is associated with an attenuated ability of IPC to prevent (endothelial) IR-injury. Interestingly, previous work in animals demonstrated that exercise training, in line with IPC, results in myocardial adaptation that allows greater recovery of cardiac function after cardiac ischaemia (Bowles et al., 1992). These cardioprotective effects against IR injury seem present after both moderate- and high-intensity training in rats (Lennon et al., 2004). Although the exact mechanisms are currently incompletely understood, and may even differ from those related to IPC, exercise may represent an alternative preconditioning stimulus that may contribute to protection against IR-injury (Thijssen et al., 2018).

Regular exercise training improves the risk against cardiovascular events (Mora et al., 2007; Green et al., 2017). Animal studies revealed that exercise training restores the attenuated efficacy of IPC in aged rat hearts (Abete et al., 2000; Wang et al., 2014). Similarly, we recently reported that lifelong exercise training is associated with increased tolerance against endothelial IR-injury (Maessen et al., 2017). Accordingly, exercise training may also attenuate endothelial IR-injury in patients with HF. In addition, the type of exercise may impact the benefits of exercise training. Michelsen et al. (2012) found that interval exercise (which shows similarities with ischaemic preconditioning in mediating repeated, short bouts of local ischaemia) induces immediate cardioprotection. Moreover, we found that a single bout of interval exercise, but not endurance exercise, protected against endothelial IR-injury in healthy young men (Seeger et al., 2015).

In the present study, we examined the effect of 12-weeks of CT or HIT on the magnitude of decline in endothelial IR-injury in HF patients. In line with recent observations (Maessen et al., 2017; Thijssen et al., 2018), we expect that exercise training will attenuate the (exaggerated) decline in endothelial function in response to IR-injury in HF patients. Moreover, based on the acute preconditioning effects of interval exercise (Michelsen et al., 2012; Seeger et al., 2015), we expect that HIT shows superior effects compared to traditional CT.

## Materials and Methods

### Subjects

A total of 29 patients (65 ± 8 years) diagnosed with HF [NYHA class II-III, history of left ventricular ejection fraction (LVEF) ≤ 45%] were included in our study for final analysis. Inclusion took place through advertisement, and via the Department of Cardiology of the Radboud University Medical Center and the Canisius-Wilhelmina Hospital (Nijmegen, Netherlands). We excluded patients who developed HF due to congenital heart disease and/or valve pathology. We excluded the following individuals who present with: diabetes mellitus (type 1 or 2), hypercholesterolemia (total cholesterol > 6.5 mmol/L), severe renal failure (glomerular filtration rate < 30 mL/min/1.73 m^2^), exercise-induced ischemia (i.e., ECG abnormalities suggestive for ischemia on maximal exercise testing), severe co-morbidities (e.g., COPD GOLD ≥ 3), pathology that restricts patients from participation to exercise (e.g., orthopedic/neurological disorders interfering with movement), pre-menopausal women or women on hormone replacement therapy, and subjects with contra-indications for maximal exercise testing (Fletcher et al., 2013). All individuals were in a stable situation, meaning that clinical and pharmacological status has not changed > 3 months prior to participation. We received ethical approval from the local Medical Ethical Committee (CMO region Arnhem–Nijmegen; Geert Grooteplein 10, 6525 GA Nijmegen, Netherlands), whilst our trial is registered in the Dutch Trial Register (NTR3671). Written informed consent was obtained before participation in this study.

### Experimental Protocol

After inclusion into our study, subjects were allocated to 12-weeks moderate-intensity CT or HIT. To control for potential changes across time, measurements were performed before and after a 12-week control period in nine HF patients unable to participate (due to transportation or time-constraints). Before and after the intervention, we examined physical fitness (using a maximal incremental cycling test) and vascular ultrasound to examine brachial artery flow-mediated dilation (FMD) before and after an IR-protocol as a surrogate for IR-injury (Kharbanda et al., 2002; Loukogeorgakis et al., 2005, 2006). Although blinding of participants was not possible, blinding of the observer during FMD analysis was applied for the allocation of the group and timing of the test. The study was originally set-up to examine the impact of both types of exercise training on clinical outcome measures (i.e., physical fitness, quality of life) and cardiac and vascular function/structure, which is published elsewhere (Benda et al., 2015a). The changes in brachial artery endothelial function after IR represented a secondary outcome measure, and were not part of the original analysis.

### Measurements

#### Subject Characteristics

Height, weight (Seca 888 Scale, Seca, Hamburg, Germany), BMI, body fat percentage (Durnin and Womersley, 1974), and waist and hip circumference were determined before and after the intervention. Heart rate and blood pressure were measured manually (WelchAllyn, Maxi-Stabil 3, Skaneateles Falls, NY, United States), whilst an electrocardiogram was used to assess cardiac rhythm. A venous blood sample was used to assess levels of fasted glucose and cholesterol.

#### Physical Fitness

Subjects performed an incremental maximal cycling test (Ergoline, Ergoselect 200k, Bitz, Germany). Subjects were instructed to pedal at a constant speed (>60 rpm) whilst workload was increased 10–15 Watt/min (dependent on sex, age, height, and previous results). Continuous breath-by-breath gas analysis was used to examine changes in oxygen uptake (LabManager V5.32.0). Peak oxygen uptake (VO_2peak_) was defined as the highest 30-s oxygen uptake during the test. We adhered to recent guidelines for the termination of the exercise test (Fletcher et al., 2013).

#### Endothelial Function

Before each experiment, participants refrained from food ingestion ≥ 6 h, caffeine and products with high levels of vitamin C ≥ 18 h, and from strenuous physical activity ≥ 24 h. Subjects were tested at the same time of day to prevent diurnal variation in FMD response. All measurements were performed in a temperature-controlled room (22.5°C) and using expert-consensus guidelines of FMD (Thijssen et al., 2011; van Mil et al., 2016). Subjects were instructed to continue medication, but to refrain from diuretics the day of testing for practical reasons. Subjects rested in a supine position with the right arm extended and immobilized, supported at an angle of ∼80° abduction from the torso. For the assessment of FMD, a rapid inflation/deflation pneumatic cuff was placed distal to the olecranon process to provide an ischaemic stimulus distal from the brachial artery to provoke vasodilation. A 10-MHz (T3000, Terason, Aloka, United Kingdom) multi-frequency linear array probe attached to a high-resolution ultrasound machine was used to perform imaging. Ultrasound parameters were set to optimize longitudinal B-mode images of the lumen/arterial wall interface. A continuous Doppler velocity assessment was obtained simultaneously, and data were collected using the lowest possible insonation angle (always < 60°), which did not vary during each study (Thijssen et al., 2011). After a resting period of > 15-min, 1-min of baseline recording of the arterial diameter and velocity was performed. Subsequently, the occlusion cuff was inflated to 220 mmHg for 5-min. The arterial diameter and velocity recordings were restarted at least 30 s before cuff deflation and continued for at least 3 min after deflation. Peak arterial diameter and flow, and the time to reach this peak after cuff deflation, were recorded. Analysis of the brachial artery diameter was performed using custom-designed edge-detection and wall-tracking software, which is independent of investigator bias. Following cuff deflation, peak diameter was automatically detected according to an algorithm as described in detail elsewhere (Black et al., 2009). Within-subject reproducibility of the FMD using this semi-automated software is 6.7–10.5% (coefficient of variation) (Thijssen et al., 2009).

#### Endothelial Ischaemia-Reperfusion

Ischaemia-reperfusion was induced by a 5-min ischaemic handgrip exercise stimulus followed by 15-min of reperfusion. Local ischaemia during handgrip exercise (rhythmic handgrip exercise at 30% of maximum handgrip strength, 1 s contraction followed by 1 s rest) was induced with upper arm cuff inflation to 220 mmHg. This ischaemic handgrip protocol leads to a (near) maximal ischaemic stimulus and peak reactive hyperaemia (Naylor et al., 2005). The transient decrease in FMD is assumed to reflect IR-induced endothelial dysfunction, a finding supported by studies that successfully mitigated this decline in FMD by well-established pharmacological (i.e., statins) and physical (i.e., ischaemic preconditioning; Kharbanda et al., 2001; van den Munckhof et al., 2013) interventions that protect against IR. Furthermore, brachial artery FMD correlates with coronary artery endothelial function in humans (Takase et al., 1998), and predicts cardiovascular events in asymptomatic subjects and in those with established cardiovascular diseases (Inaba et al., 2010; Ras et al., 2013). This model, therefore, is a frequently used and surrogate endpoint for IR-injury (Kharbanda et al., 2001; van den Munckhof et al., 2013).

### Exercise Training

Supervised exercise training was performed in a rehabilitation/hospital setting (twice a week). All missed exercise sessions were replaced to ensure a 100% compliance. Warm-up consisted of 10-min at 40% of maximal workload (W_max_) and concluded with a 5-min cool-down at 30% W_max_. Workload was gradually increased across the training period. CT consisted of 30-min at 60–75% W_max_, aiming at a Borg score of 12–14 (Piepoli et al., 2011). HIT consisted of 10 periods of intervals of 1-min at 90% W_max_ followed by 2.5-min at 30% W_max_, aiming at a Borg score of 15–17 during the high-intensity intervals. Control subjects were instructed not to alter their daily physical activities. A frequency of twice a week was adopted to match the exercise training regimes typically adopted in cardiac rehabilitation to ensure that the observations from our study can be more easily translated to daily route in HF management.

### Statistical Analysis

We have made a pre-study sample size estimation based on previous studies examining the difference in effect between CT and HIT. No previous study examined the impact of exercise training on endothelial IR. We therefore based our estimations on previous studies examining the impact of exercise training on vascular function measured using the FMD. Some studies suggest *n* = 2–3 per group is sufficient (Wisloff et al., 2007; Fu et al., 2013), whilst data from others suggest several thousand subjects must be recruited to detect differences between CT and HIT (Iellamo et al., 2013). We rationalized that *n* = 10–20 will provide (clinically) meaningful insight into the effect of exercise training. Therefore, we aimed for *n* = 20 for both exercise training groups (and *n* = 10 in the control group).

Data was analyzed using SPSS Statistics 20.0 (IBM Corp., Armonk, NY, United States). Parameters were checked for normality using a Kolmogorov–Smirnov test. When data was not normally distributed, a non-parametric alternative was used or natural logarithmic data transformation was applied. Categorical and nominal parameters were compared with a Chi-Square test. Baseline characteristics of the groups were compared with a one-way ANOVA or Kruskal–Wallis test when data was not normally distributed. Data are presented as mean ± standard deviation (SD), unless stated otherwise. Significance level was set at *P* < 0.05.

To examine the impact of exercise training (“time”: pre vs. post) and the type of exercise (“type”: CT vs. HIT) on the change in FMD after IR (“IR”: baseline vs. post-IR), we adopted a linear mixed model analysis. To control for the potential impact of within- and between-subject differences in baseline diameter on FMD (Atkinson and Batterham, 2013), we used logarithmically transformed diameter data included baseline arterial diameter as a covariate within the linear mixed model analysis. For aim 1, FMD was analyzed with random factor subject and 2 fixed factors: time (pre vs. post) and IR (baseline FMD vs. post-IR FMD). When a significant interaction-effect was found, we adopted *post hoc* analysis to identify differences. To examine whether the type of exercise impacted the effect of exercise training (i.e., aim 2), we repeated this analysis with the addition of “type” (CT vs. HIT) as a fixed factor.

## Results

Out of the 59 individuals who were screened for this study, 15 HF patients did not meet the inclusion criteria and 11 patients declined participation (10 due to time constraints, 1 due to illness). Twenty-four individuals were randomly assigned to HIT or CT, whilst 9 HF patients were included as controls (non-randomized). No drop-outs were observed in the control group. A total of 4 drop-outs were present (71 ± 2 years; male:female 3:1; NYHA class II:III 3:1). In both exercise training groups 1 person dropped out because clinical progression and 1 due to musculoskeletal complaints. Except for sex (i.e., more females in the control group), we found no differences between groups in body characteristics (e.g., age, BMI) or clinical status (e.g., NYHA-class, etiology, blood pressure, LVEF, physical fitness) (Table 1).

**Table 1:** Subject characteristics and cardiovascular medication.

	CT (*n* = 10)	HIT (*n* = 10)	Control (*n* = 9)	*P*-value
Age (years)	64 ± 8	63 ± 8	67 ± 7	0.57
Sex (male:female)	10:0^∗^	9:1	5:4	0.028
Body mass index (kg/m^2^)	28.9 ± 4.7	28.1 ± 7.5	25.4 ± 2.7	0.24
NYHA class (II:III)	8:2	8:2	8:1	0.84
Etiology (Isch:Non-isch)	8:2	7:3	5:4	0.51
Systolic blood pressure (mmHg)	132 ± 23	132 ± 18	130 ± 25	0.98
Diastolic blood pressure (mmHg)	83 ± 11	79 ± 10	78 ± 14	0.48
VO_2peak_^†^ (mL/min/kg)	21.0 ± 3.4	19.1 ± 4.1	17.4 ± 5.8	0.26
VO_2peak_^†^ (% of predicted VO_2peak_)	86 ± 8	79 ± 17	81 ± 22	0.63
LVEF (%)	38 ± 6	37 ± 6	40 ± 11	0.84
Medication				
Angiotensin converting enzyme-inhibitors	5 (50%)	6 (60%)	8 (89%)	0.19
Angiotensin II receptor antagonists	4 (40%)	4 (40%)	1 (11%)	0.30
Aldosterone antagonist	6 (60%)	7 (70%)	8 (89%)	0.36
Diuretics (loopdiuretics)	7 (70%)	6 (60%)	4 (44%)	0.50
β-blockers	10 (100%)	9 (90%)	9 (100%)	0.37
Antiplatelet drugs	6 (60%)	4 (40%)	3 (33%)	0.47
Coumarin derivates	4 (40%)	7 (70%)	4 (44%)	0.35
Statins	10 (100%)	9 (90%)	4 (44%)^§^	0.007

*Data is presented as mean ± SD. *P*-values refer to a one-way ANOVA. ^†^Data was unavailable for 1 subject in the CT-group and 3 subjects in the control-group. ^∗^Significantly less females compared to the control-group. ^§^ Lower compared to CT-group and HIT-group.*

### Exercise Training

Exercise intensity of CT was 66 ± 5% of maximal workload. The intervals during HIT were performed at 102 ± 7% of maximal workload (*P* < 0.001). Relating average exercise intensity across the entire exercise bout to individual peak heart rates, we found that CT was performed at 81 ± 7% and HIT at 83 ± 9% of maximum HR (*P* = 0.70). Subjective exercise intensity measured using Borg-scores revealed no difference between CT and HIT (13 ± 1 and 14 ± 1, respectively, *P* = 0.27). We found no significant increase in physical fitness after training when presented as VO_2peak_, whilst a significant increase was found when presented as percentage of the predicted VO_2peak_ (Table 2). For both parameters, no differences were found between groups (both *P* = 0.08, Table 2). Maximum workload improved after CT and HIT (both *P* < 0.001), whilst these changes also did not significantly differ between groups (*P* = 0.07, Table 2).

**Table 2:** Maximal incremental cycling test.

	CT (*n* = 10)		HIT (*n* = 10)		*P*-value		
	Pre	Post	Pre	Post	Time	Type	Time^∗^Type
VO_2peak_ (mL/min)	1881 ± 214	1887 ± 27	1662 ± 562	1792 ± 559	0.06	0.44	0.08
VO_2peak_ (% pred. VO_2peak_)	86 ± 8	87 ± 10	79 ± 17	85 ± 16	0.044	0.48	0.08
Max. workload (Watt)	145 ± 22	152 ± 26	126 ± 38	142 ± 45	< 0.001	0.24	0.07

*Data is presented as mean ± SD. *P*-values refer to a two-way repeated measures ANOVA between the two training groups. One subject in the CT-group did not reach VO_*2peak*_, and therefore only VE/VCO_*2*_ slope and VO_*2*_ at AT could be determined.*

### Endothelial Ischaemia-Reperfusion Injury

**FIGURE 1:**
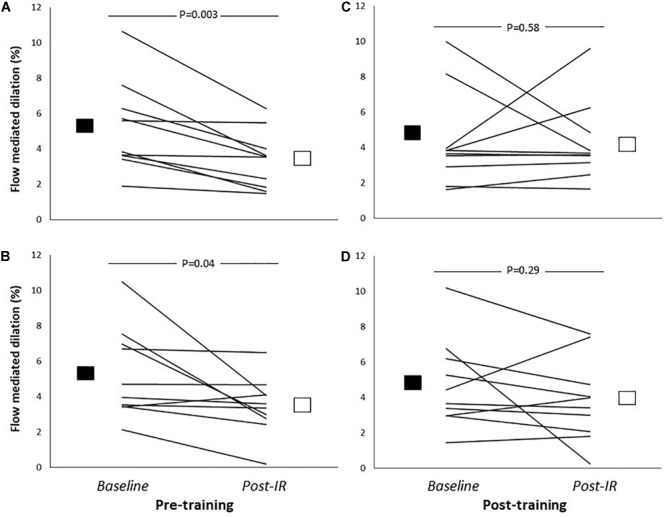
Brachial artery flow-mediated dilation (relative change from resting diameter, %) before (Baseline; solid square) and after 5-min of ischaemic handgrip exercise and 20-min reperfusion (Post-IR; open square) before and after 12-week continuous training (**A,B**: *n* = 10) or high-intensity interval training (**C,D**: *n* = 10). *P*-values refer to *post hoc* analysis related to the impact of IR-injury (LMM effect of IR: *P* = 0.01). Lines represent individual data for all participants.

Prior to training, brachial FMD significantly declined in response to IR (Table 3). Exercise training in HF patients did not alter brachial artery FMD (Table 3 and Figure 1). *Post hoc* analysis revealed that after 12-week exercise training, IR did not change FMD (Figure 1).

**Table 3:** Brachial artery flow-mediated dilation before and after ischaemia-reperfusion injury prior to and after 12-week exercise training.

	Continuous training (*n* = 10)				High-intensity training (*n* = 10)						
	Pre-training		Post-training		Pre-training		Post-training		LMM		
CT (*n* = 10)	Baseline	Post-IR	Baseline	Post-IR	Baseline	Post-IR	Baseline	Post-IR	IR	Training	IR^∗^training
Diameter (mm)	4.5 ± 0.5	4.6 ± 0.5	4.5 ± 0.5	4.5 ± 0.4	4.4 ± 0.9	4.4 ± 0.8	4.4 ± 0.8	4.5 ± 0.6	0.63	0.91	0.99
FMD (%)	5.2 ± 2.5	3.4 ± 1.6*	4.8 ± 3.0	4.2 ± 2.3	5.3 ± 2.6	3.5 ± 1.6*	4.7 ± 2.5	3.8 ± 2.3	0.013	0.90	0.30
FMD_n_ (%)	5.2 ± 2.4	3.3 ± 1.6*	4.8 ± 2.8	4.2 ± 2.2	5.3 ± 2.4	3.5 ± 1.6*	4.7 ± 2.4	3.8 ± 2.3	0.016	0.91	0.28
SR_AUC_ (A.U., 10^3^)	19.9 ± 9.6	20.1 ± 8.2	18.6 ± 7.4	20.6 ± 8.8	17.8 ± 9.2	22.4 ± 9.1	22.3 ± 7.7	17.8 ± 6.5	0.76	0.89	0.33

*^∗^*Post hoc* significantly different from baseline at *P* < 0.05.*

Statistical analysis revealed no significant differences in baseline FMD or in the magnitude of decline in FMD in response IR between the CT and HIT groups (Table 2). Furthermore, no significant differences were found for the impact of the type of training (i.e., CT vs. HIT) on baseline FMD (“time^∗^type”-interaction, *P* = 0.81) or for the magnitude of decline in FMD post-IR (“time^∗^type^∗^IR”-interaction, *P* = 0.99).

### Control Group

The control group showed no change in VO_2peak_ (1.36 ± 0.56 vs. 1.39 ± 0.60 L, *P* = 0.50) or predicted VO_2peak_ (81 ± 22 vs. 82 ± 21%, *P* = 0.74) over 12 weeks without additional training. We found no change in baseline brachial artery FMD or in the magnitude of decline in brachial artery FMD after IR (Table 4).

**Table 4:** Brachial artery flow-mediated dilation before and after ischaemia-reperfusion injury prior to and after 12-week control period (*n* = 9).

	Before		After		LMM		
	Baseline	Post-IR	Baseline	Post-IR	IR	Time	IR^∗^time
Diameter (mm)	4.1 ± 0.8	4.1 ± 0.8	4.0 ± 0.8	4.1 ± 0.9	0.89	0.93	0.85
FMD (%)	5.3 ± 2.0	3.9 ± 2.2*	5.4 ± 2.4	3.6 ± 1.7*	0.03	0.93	0.77
FMD_n_ (%)	5.2 ± 1.9	3.9 ± 2.2*	5.4 ± 2.2	3.6 ± 1.7*	0.006	0.80	0.84
SR_AUC_ (A.U., 10^3^)	17.7 ± 13.4	19.6 ± 10.8	22.5 ± 15.5	23.5 ± 8.2	0.72	0.29	0.91

*^∗^*Post hoc* significantly different from baseline at *P* < 0.05.*

## Discussion

This study is the first study in humans to examine whether regular exercise training affects the exaggerated endothelial IR-injury observed in HF patients. Our study provides the following observations. First, we found that 12-weeks of exercise training in HF patients attenuated the magnitude of endothelial IR-injury in HF patients, whereas these beneficial effects are not accompanied by an improvement in baseline endothelial function. Second, the ability of exercise training to attenuate endothelial IR-injury is independent on the type of exercise training in HF patients. Our data, therefore, suggest that both types of exercise training improve tolerance of the vasculature against local ischaemia within 12-weeks. Supported by the presence of exaggerated endothelial IR-injury in HF (Ferdinandy et al., 2014; Seeger et al., 2016), but also by the inability of (non)pharmacological interventions to improve these responses (Heusch, 2013), future studies are warranted to further explore the potential meaning and relevance of the ability of exercise training to attenuate endothelial IR-injury in HF patients.

Previous work from both animal and human studies have provided increasing evidence that exercise possesses preconditioning effects (Thijssen et al., 2018). Immediate and chronic protective effects of exercise training have been reported, in that a smaller or even abolished decline in endothelial IR-injury is reported in response to acute exercise in healthy young (Seeger et al., 2015) and in physically active older humans (Maessen et al., 2017). To further explore this field, our observation represents the first in the literature in humans that examined and showed that regular exercise training is able, within subjects, to improve tolerance against endothelial IR-injury. Interestingly, these effects were present without changes in baseline FMD, as also described in our previous work (Benda et al., 2015a). The lack of improvement in endothelial function after exercise training in HF patients is not in line with a majority of previous work (Green et al., 2017). Potential explanations for this relate to the relatively low volume and/or frequency of exercise training in our study. Alternatively, explanations relate to the relatively high baseline FMD prior to training [and thus less potential for improvement (Green et al., 2014b)] and/or attenuated shear rate responses during training in HF patients (providing a smaller stimulus for vascular adaptation; Benda et al., 2015b). At least, our observations suggest that benefits of exercise on the vasculature may be mediated through various pathways, including tolerance against potentially harmful stimuli.

The observations from our study raise questions related to the potential mechanisms underlying these observations. Based on the anti-atherogenic characteristics and vasodilator effects of NO, this molecule may contribute to increased tolerance against ischaemia. Indeed, infarct-sparing effects of training were abolished in eNOS-deficient mice (de Waard et al., 2010) and in hearts of trained rats when treated with eNOS inhibitors (Farah et al., 2013). However, we found no changes in brachial artery FMD, a measure that reflects NO-mediated vasodilator function (Green et al., 2014a). An alternative explanation relates to the ATP-sensitive potassium channels, especially since opening of these channels before IR-injury may protect the heart (Powers et al., 2014). To support this idea, exercise training in animals resulted in a smaller infarct size, whilst sarcoK_ATP_-blockade, but not mitoK_ATP_-blockade, abrogated the protective effect (Brown et al., 2005). Since exercise training improves mitochondrial function (Powers et al., 2014) this may contribute to increased tolerance against ischaemia by virtue of the expression of (antioxidant) proteins to minimize ROS formation. Finally, training may affect opioid- and/or adenosine-receptors, especially since the infarct-sparing effects of exercise training can be prevented by blocking (delta) opioid (Michelsen et al., 2012) or adenosine receptors (Domenech et al., 1998).

The second aim of our study was to examine whether the type of exercise affected the effects of exercise training. Based on earlier observations that high-intensity interval exercise has obvious similarities with IPC (i.e., repeated periods of local hypoxia or relative deoxygenation), but also because a single bout of interval but not endurance exercise prevents endothelial IR-injury (Seeger et al., 2015), we expected HIT to lead to superior effects compared to continuous exercise training. Despite these potential differences with acute bouts of exercise, we found that the type of exercise training did not alter our main outcomes. Interestingly, in a previous study it was found that regular resistance training is associated with less endothelial IR-injury in young subjects (DeVan et al., 2011). In another study, lifelong regular endurance exercise training was associated with protection against endothelial IR-injury in an older population (Maessen et al., 2017), a finding also observed by others (Devan et al., 2011). Taken together, our study provides further support for the ability of regular exercise training to attenuate endothelial IR-injury in humans, even in those with cardiovascular disease, whereas the type of exercise training seems less important to achieve these benefits.

Previous work found exaggerated endothelial IR-injury in this population, but also attenuated efficacy of ischaemic preconditioning (Seeger et al., 2016). In line with these findings, no clinical benefit of ischaemic preconditioning interventions have been observed in clinical studies (Ferdinandy et al., 2014). Nonetheless, exercise training was effective in attenuating IR-injury. First, this suggests that the exaggerated decline in endothelial IR-injury is not the result of HF *per se*. Secondly, our observations raise the hypothesis that exercise may represent a more powerful preconditioning stimulus compared to ischaemic preconditioning. Exercise more rapidly induces hypoxia (and across a larger tissue area) compared to repeated cuff inflation around an arm. This could translate to clinically relevant effects, especially since exercise (preconditioning) is easier and more frequently to perform compared to ischaemic preconditioning. Taking these differences into account, exercise may represent a more feasible and effective preconditioning strategy for clinical use than traditional ischaemic preconditioning using blood pressure cuffs.

### Limitations

An obvious limitation of our study it the inability to translate our model of endothelial IR-injury to cardiac tissue and/or (non)fatal tissue damage. Nonetheless, this model is frequently used, whilst results related to preconditioning stimuli match observations from both pre-clinical and clinical observations. Nonetheless, future work is required to better understand these effects and improve translation (and mechanistic insight) of our results. Another limitation of our study is the relatively small number of patients. Nonetheless, our sample size was not different from other studies examining the impact of (various types of) exercise training (Wisloff et al., 2007; Fu et al., 2013; Ramos et al., 2015). Importantly, we have adopted state-of-the-art techniques for and followed expert-consensus guidelines to assess vascular function. Although our sample size may have been insufficient to detect differences between the two types of training, previous work also suggested that moderate- and high-intensity training lead to comparable recovery of cardiac function after cardiac ischaemia (Lennon et al., 2004).

## Conclusion

Our results reveal that 12-weeks exercise training in HF patients leads to attenuation in endothelial IR injury, an effect that is independent of the type of exercise training (continuous endurance vs. high-intensity interval). Although exercise training did not alter resting endothelial function, our data indicate that regular exercise training improves tolerance against endothelial IR injury. These changes may contribute to the cardioprotective effects of exercise training. Moreover, in light of the disappointing results from clinical studies adopting ischaemic preconditioning, exercise preconditioning may be a more potent, but also easier and freely applicable, stimulus for cardioprotection in humans.

## Data Availability

The datasets generated for this study are available on request to the corresponding author.

## Author Contributions

NB, JS, AvD, MH, and DT conceived and designed the research. NB, JS, and AvD contributed to recruitment of participants. NB, JS, AvD, and DT acquired and analyzed the data. NB, AvD, MH, and DT interpreted results of the research. All authors edited and revised the manuscript, and approved final version of the manuscript.

## Conflict of Interest Statement

The authors declare that the research was conducted in the absence of any commercial or financial relationships that could be construed as a potential conflict of interest.
